# Polymorphisms in the ACE I/D (*rs*4646994) and ACE2 G8790A (*rs*2285666) in Young Children Living in the Amazon Region and SARS-CoV-2 Infection

**DOI:** 10.3390/tropicalmed9110270

**Published:** 2024-11-07

**Authors:** Yan Cardoso Pimenta, Flávia Freitas de Oliveira Bonfim, Carlos Eduardo da Silva Figueiredo, Bruno Loreto de Aragão Pedroso, Mauro França Silva, Alberto Ignacio Olivares Olivares, Isabella Fernandes Delgado, José Paulo Gagliardi Leite, Marcia Terezinha Baroni de Moraes

**Affiliations:** 1 Laboratory of Comparative and Environmental Virology, Oswald °Cruz Institute, Oswald °Cruz Foundation, Fiocruz, Avenida Brasil, 4365, Manguinhos, Rio de Janeiro 21040-360, RJ, Brazil; yancpimenta@gmail.com (Y.C.P.); flaviabiomar@gmail.com (F.F.d.O.B.); cesfigueiredo@gmail.com (C.E.d.S.F.); bruno.loreto.aragao@hotmail.com (B.L.d.A.P.); mauro@bio.fiocruz.br (M.F.S.); jpgleite@ioc.fiocruz.br (J.P.G.L.); 2 Post-Graduate Program in Sanitary Surveillance, National Institute for Quality Control in Health, Oswald °Cruz Foundation, Fiocruz, Avenida Brasil, 4365, Manguinhos, Rio de Janeiro 21040-360, RJ, Brazil; isabella.delgado@fiocruz.br; 3 Post-Graduate Program in Tropical Medicine, Oswald °Cruz Institute, Oswald °Cruz Foundation, Fiocruz, Avenida Brasil, 4365, Manguinhos, Rio de Janeiro 21040-360, RJ, Brazil; 4 National Institute of Women, Children and Adolescents’ Health Fernandes Figueira, Oswald °Cruz Foundation (Fiocruz), Avenida Rui Barbosa, 716-Flamengo, Rio de Janeiro 22250-020, RJ, Brazil; 5 Technological Coordination, Tetraviral Vaccine, Immunobiological Technology Institute (Biomanguinhos), Oswald °Cruz Foundation, Fiocruz, Avenida Brasil, 4365, Manguinhos, Rio de Janeiro 21040-360, RJ, Brazil; 6 Secretaria Estadual de Saúde de Roraima, SESAU/RR, Rua Madrid, 180-Aeroporto, Boa Vista 69310-043, RR, Brazil; albertouffr@gmail.com; 7 Medicine & Health School, State University of Roraima, Rua Presidente Juscelino Kubitscheck, 300, Canarinho, Boa Vista 69360-000, RR, Brazil

**Keywords:** COVID-19, SARS-CoV-2, susceptibility, angiotensin-converting enzyme

## Abstract

COVID-19 infection caused by SARS-CoV-2 continues to cause significant mortality and morbidity. ACE2 is a key regulator of the renin–angiotensin–aldosterone system (RAAS). Differences in COVID-19 severity are thought to be due to the imbalance of RAAS/ACE mutations. This retrospective study evaluated the detection and genetic susceptibility to SARS-CoV-2 infection in 202 children ≤3 years of age living in the Amazon region in 2021. The angiotensin-converting enzyme ACE I/D (*rs*4646994) and ACE2 G8790A (*rs*2285666) polymorphisms were detected by SYBR GREEN real-time PCR and PCR-RFLP/*Alu*l digestion, respectively. SARS-CoV-2 detection was performed by RT-qPCR in feces and saliva samples collected simultaneously from the same children presenting acute gastroenteritis (AGE) or acute respiratory infection (ARI). The frequency of SARS-CoV-2 detected by qRT-PCR in children was low (5.9%, 12/202), although higher in the group of children with AGE (8.9%, 9/101) than with ARI (2.9%, 3/101). Susceptibility to SARS-CoV-2 infection was not verified due to the low frequency. Homozygous II (*rs*4646994) children were the majority (87.1%, 176/202). Boys with genotype A (*rs*2285666) were more susceptible to ARI and pneumonia symptoms than AGE (OR = 3.8, 95% CI 1.4–10.3, *p* 0.007). Boys with genotype G (*rs*4646994) or the combination II + G were more susceptible to acquiring AGE. Surveillance, along with understanding their causes, is crucial to controlling ARI and COVID-19 in children living in low-income countries.

## 1. Introduction

Brazil has had one of the highest pediatric COVID-19 mortality rates during the pandemic [1,2,3]. The COVID-19 disease caused by Coronavirus 2 (SARS-CoV-2) is characterized by symptoms that include high fever, chills, coughing, and shortness of breath or difficulty breathing. It may also present other symptoms, such as diarrhea, myalgia, fatigue, expectoration, and hemoptysis [4]. Acute gastroenteritis (AGE) causes high morbidity and mortality in children ≤5 years of age, especially in regions of low income. Symptoms of AGE have been associated with COVID-19, although less common than respiratory symptoms [5,6,7,8,9].

The angiotensin-converting enzyme (ACE2) is a key regulator of the renin–angiotensin–aldosterone system (RAAS). ACE2 is the cell membrane receptor of SARS-CoV-2, mediating viral entry into cells [10,11,12]. Epithelial cells of the small intestine containing ACE2 receptors bind to SARS-CoV-2, causing gastrointestinal symptoms [8,9]. SARS-CoV-2 causes ACE/ACE2 balance disruption and RAAS activation, which ultimately leads to COVID-19 progression [10,11,12,13].

Several potentially functional gene polymorphisms have been identified in *ACE* and *ACE*2 genes. Associations between these polymorphisms and human disorders have been assessed in different populations. Two of these polymorphisms are described below and associated with greater host susceptibility to SARS-CoV-2 [14,15,16]. The insertion/deletion polymorphism of the *ACE* gene is defined as either the presence (insertion, I) or absence (deletion, D) of a 287 base pair inserted in intron 16 of the gene in chromosome 17q23 [17]. It is commonly referenced as *rs*4646994. Several pulmonary diseases and their activity are associated with elevated levels of *ACE* gene activity, including sarcoidosis, hypoxic pulmonary hypertension, idiopathic pulmonary fibrosis, and acute respiratory distress syndrome (ARDS). The insertion appears to reduce ACE expression; thus, DD homozygotes have 65% more, and ID heterozygotes have 31% more ACE than II homozygotes [18]. Studies indicate that the overall frequency of the D allele is 54%, unrelated to gender but connected to ethnic differences. DD homozygous individuals are associated with the highest ACE levels in most ethnic groups, and the ID heterozygous shows the greatest variation (47%) in ACE levels [19]. The *ACE*2 gene is located in the X chromosome in the human genome. ACE2 is mainly expressed in the cardiovascular, renal, and gastrointestinal tissues. Moreover, ACE2 has also been found in the brain, lungs, and testis. A splice region variant named *rs*2285666 (G > A, Intron 3/4) is the best-described single nucleotide polymorphism (SNP) in the *ACE*2 gene. Several reports have revealed correlations with diabetes, cerebral stroke, coronary heart disease, and hypertension. The A genotype is linked to higher ACE2 levels in the serum of healthy individuals. Increased ACE2 receptor expression in membranes has been reported in men, in populations with age above 60, patients with metabolic disorders like diabetes and hypertension, and patients with respiratory or cardiac diseases. Interestingly, these conditions are also risk factors for a severe course of COVID-19 because the ACE2 acts as a cellular receptor for virus entry [16,20,21,22,23].

This study was carried out with children ≤3 years of age living in the Amazon region who attended emergency care at the “Hospital da Criança de Santo Antônio (HCSA)”, Brazil. All children selected for this study have physical characteristics of indigenous ethnicities. For each child with acute respiratory infection (ARI) selected for sample collection, another child with acute gastroenteritis (AGE) was also selected. The SARS-CoV-2 detection profile was verified in samples from children with ARI and AGE. Susceptibility to SARS-CoV-2 infection was analyzed based on the profile of the ACE polymorphisms selected here.

## 2. Materials and Methods

### 2.1. Sampling

Collections were carried out from May to June 2021 at the HCSA, located in the capital of Boa Vista in the Brazilian state of Roraima (RR). The HCSA exclusively receives children ≤12 years old, as previously described [24]. All children in this study were treated in the HCSA emergency care. A total of 202 fecal and 202 saliva samples (collected at least 1 h before or after breastfeeding) were collected in parallel to 202 children ≤3 years of age (one fecal/saliva sample/child): 101 with AGE and 101 with ARI (inclusion criteria according to the World Health Organization (WHO)) [25]. The pediatrician participating in this study collected all samples immediately after the children’s admissions and examined them; their parents or guardians were interviewed for data collection and completion of a form containing clinical and epidemiological information for each child. Immediately after collection, all samples were temporarily stored at −20 °C in the HCSA and then sent together with clinical–epidemiological records to the Laboratory of Comparative and Environmental Virology–Regional Rotavirus Reference Laboratory (LVCA-RRRL) under strict transport criteria to maintain the temperature and integrity of the samples. The LVCA-RRRL is part of the ongoing national network for AGE surveillance and is coordinated by the General Coordination of Public Health Laboratories, Brazilian Ministry of Health.

### 2.2. SARS-CoV-2 Detection

All samples were strictly kept under −20 °C until the moment of processing. Then, 10% fecal suspensions in pH 7.4 phosphate-buffered saline (PBS) solutions were prepared. The saliva samples were processed using 1.2 mL of PBS to a final dilution of 1:5. Total viral nucleic acid (feces and saliva) and total genome DNA (saliva) were obtained as previously described [24]. Total nucleic acids were stored at under −80 °C. Quantitative Reverse Transcription Polymerase Chain Reaction (qRT-PCR) was performed for detection of SARS-CoV-2 *E*, *N* and *RdRp* genes separately as monoplexes with primers and probes according to the Laboratório de Vírus Respiratórios e Sarampo, COVID-19 National Reference Laboratory of Brazil, Fundação Oswald °Cruz (Fiocruz), and WHO protocols [26]. All the reactions were conducted with the Applied Biosystems 7500 Real-Time PCR System (Applied Biosystems, Carlsbad, CA, USA) using 5 µL of total viral nucleic acid extracted (feces and saliva) and the SuperScript™ III PlatinumTM One-Step qRT-PCR (Invitrogen, Carlsbad, CA, USA) kit, according to the manufacturer’s recommendations. All samples that showed signals of at least one of the three genes (*E*, *N*, or *RdRp*) crossing the threshold line in both replicas up to a threshold cycle (Ct) value of 40.00 that also presented a characteristic sigmoid curve were regarded as positive and transformed into viral load in copies per milliliter. Negative and positive control samples (Total RNA from feces, saliva, and nasopharyngeal swab samples, extracted in parallel with the tested samples) were obtained from the biorepository bank of the LVCA-RRRL, Oswald °Cruz Institute, Fiocruz.

### 2.3. Genotyping ACE rs4646994 and ACE2 rs2285666 of Polymorphisms

Polymorphism ACE *rs*4646994 was verified by SYBR Green Real-Time PCR according to the previously described modified protocol [14,27] using the SYBR™ Green PCR Master Mix (Thermo Fisher Scientific, Waltham, MA, USA), following the manufacturer’s instructions. Amplification and detection were performed with the ABI PRISM^®^ 7500 Sequence Detection System (Applied Biosystems, Carlsbad, CA, USA) using 5 µL of total viral nucleic acid extracted from saliva. All reactions were performed in duplicates, and the PCR temperature cycling conditions and analysis of melting peaks for I and D alleles were carried out as previously described [14,27]. The ACE2 *rs*2285666 was detected by restriction fragment length polymorphism PCR (PCR-RFLP), as previously described [14,28], using iTaq™ DNA Polymerase (Bio-Rad Laboratories, Hercules, CA, USA) and *Alu*I (Invitrogen, Carlsbad, CA, USA), according to manufacturer instructions. After digestion, the 281 and 185 bp fragments identifying the A allele and 466 base pair (bp) band identifying G were visualized with electrophoresis on SYBR™ green I stained (Invitrogen, Carlsbad, CA, USA) agarose, Low Melting Point (LMP), preparative grade for small fragments (Promega, Madison, WI, USA).

### 2.4. Confirmation of ACE rs4646994 and ACE2 rs2285666 Polymorphisms by Sanger Nucleotide Sequencing

PCR amplicons were obtained using primers used in the SYBR Green Real-Time PCR (*rs*4646994) and PCR-RFLP (*rs*2285666) methods, respectively. Together with the same primers, the obtained amplicons were sent to “ACT Gene—Análises Moleculares” (Porto Alegre, Brazil) for Sanger sequencing.

### 2.5. Statistical Analysis of Data, Maps and Nucleotide Analysis

The Statistica 12.6 software was used for all statistical analysis. The statistical tests were Pearson Chi-Square (differences were considered statistically significant at *p* > 0.05). Odds ratio (OR) values were calculated according to Hoppe et al. 2008 [29] verified at https://www.medcalc.org/calc/odds_ratio.php (accessed on 21 October 2024). The electropherograms of the *ACE* gene nucleotide sequences were analyzed using the free tracer viewer Chromas 2.4 (Technelysium Pty Ltd., South Brisbane, Australia). ACE *rs*4646994 and ACE2 *rs*2285666 polymorphisms were verified using the Mega Molecular Evolutionary Genetic Analysis Version X software and compared with reference nucleotide sequences according to how it was previously described [27,28]: available on the GenBank database of National Center for Biotechnology Information (NCBI). The *ACE* gene polymorphisms identified so far were accessed through the NCBI database of SNPs (https://www.ncbi.nlm.nih.gov/snp; accessed on 21 October 2024).

## 3. Results

### 3.1. Clinical and Epidemiological Characteristics of Children Living in the Amazon Region and Samples

The children in this study live in Brazil, in 13 of the 15 municipalities of the state of RR. The number of children by municipality and ethnic group is presented in the following Table 1.

Most children live in indigenous communities located in the municipality of Boa Vista (75.7%, 151/202); the indigenous ethnicities that live in these indigenous communities are Macuxi, Wapixana, Taurepangue, and Yanomami. The detected frequencies of boys and girls with AGE (respectively, 51.2%, 63/121 and 46.9%, 38/81) were like those detected with ARI (respectively, 47.9%, 58/121, and 53%, 43/81). All samples were collected no more than 3 days after symptom onset. The main clinical symptom presented by all children in this study was fever (>38.5 °C): 93% (94/101) in children with AGE and 89.1% with ARI. Mucus in feces was much more common in children with AGE (64.3%, 65/101) than with ARI (1%, 1/101). Coughing was more common in children with ARI (87.1%, 88/101) than with AGE (21.8%, 22/101). Vomiting and abdominal pain were more frequent in children with AGE (respectively, 59.4%, 60/101, and 97%, 98/101) than with ARI (respectively, 29.7%, 30/101, and 63.3%, 64 /101). Bronchiolitis and pneumonia were diagnosed only in children with ARI (88.1%, 89/101). For each child (*n* = 202) with AGE or ARI, two samples were collected in the months of May (35.6%; 144/404), June (56.5%; 228/404), and July (7.9%; 32/404).

### 3.2. SYBR Green Real-Time PCR to ACE rs4646994 and PCR-RFLP/Alul Digestion to ACE2 rs2285666 as Detection Polymorphisms Methods

**Figure 1:**
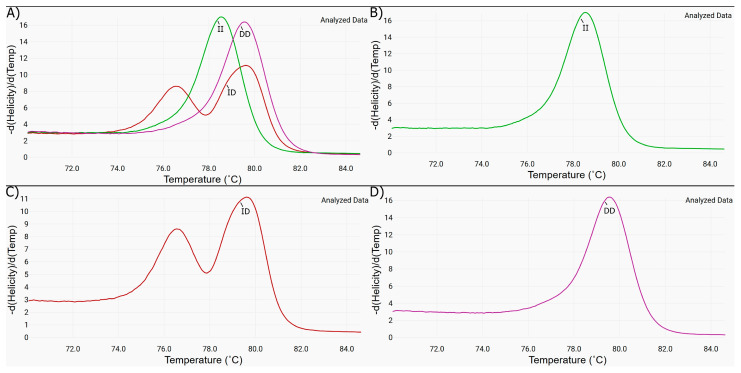
(**A**–**C**) The figure shows the electropherograms obtained from total DNA extracted from saliva samples of children with different ACE *rs4646994* polymorphisms: (**A**) overlapping of three peaks to II, ID, and DD genotypes, according to the melting temperature of 69 °C for II and 74 °C for DD; (**B**) II homozygous; (**C**) ID heterozygous; and (**D**) DD homozygous.

Figure 1 presents the electropherograms corresponding to the ACE *rs*4646994 polymorphism obtained from the total genomic DNA (extracted from saliva samples) of each child in this study. All obtained electropherograms were like those described by Lin et al., 2001 [27], according to the melting temperature for I/D polymorphisms. The polymorphism profile for all children presenting AGE or ARI can be determined (100%, 202/202). The identification of the ACE2 *rs*2285666 polymorphism, using PCR-RFLP/*Alu*l digestion according to the method described by Benjafield et al. 2004 [28], yielded 86% (87/101) genotyping for children with AGE and 77% (78/101) for children with ARI (total 81.7%; 165/202). Samples with insufficient amount of genomic DNA (18.3%; 37/202) presented unclear identification by RFLP and could not be considered. A total of 10% of amplicons obtained with primers used in SYBR Green real-time PCR and PCR-RFLP/*Alu*l digestion methods, with genomic DNA from the children in this study, were sent for Sanger sequencing, confirming the results obtained as described here.

### 3.3. Boys with the ACE2 rs2285666 Have Genetic Susceptibility to Acute Respiratory Infection/Pneumonia

Homozygous II children (ACE *rs*4646994 polymorphism) with AGE or ARI (*n* = 202) presented similar frequencies (89.1%, 90/101 for AGE; 85.1%, 86/101 for ARI) and were the predominant genotype in relation to the ID heterozygous (4.9%, 5/101 for AGE; 9.9%, 10/101 for ARI) and DD homozygous (5.9%, 6/101 for AGE; 5.0%, 5/101 for ARI) children (Table 2).

Regarding ACE *rs*2285666, 28.6% (18/63) of boys with AGE had the A genotype, and 58.7% (37/63) had the G genotype. The frequencies of A and G genotypes for boys with ARI were similar, corresponding to 37.9% (22/58) and 34.5% (20/58), respectively. Girls with AGE or ARI were, respectively, AA homozygous at 15.8% (6/38) and 18.6% (8/43); AG heterozygous at 28.9% (11/38) and 16.3% (7/43); and GG homozygous at 39.5% (15/38) and 48.8% (21/43) (Table 2).

The probability of occurrence of AGE or ARI in the children, according to the genotypes of the *ACE* gene polymorphisms selected in this study, or whether the group was girls or boys, was verified by calculating the Odds ratio (Table S1A–E, Supplementary Material and Table 3). Considering the association of the ACE2 *rs*2285666 polymorphism with AGE or with ARI (boys or girls as control groups), it was verified that boys presenting ARI (*n* = 58) with A genotype *(n* = 22) were more susceptible to ARI (OR = 3.8, 95% CI 1.4 to 10.3; *p* 0.007) than to AGE (OR = 2.1, 95% CI 0.7 to 6.0; *p* 0.1) compared to girls (ARI as a bad outcome for girls; OR = 0.2, 95% CI 0.09 to 0.7; *p* 0.007; AGE as a bad outcome for girls; OR = 0.4, 95 % CI 0.1 to 1.3; *p* 0.1) (Table S1A, Supplementary Material). Most boys with ARI were also diagnosed with pneumonia (81.8%, 18/22). However, no statistical significance was observed when comparing all children (boys and girls with both AGE and ARI) with respect to the specific symptoms of pneumonia (Table 3). The susceptibility of boys to ARI was confirmed for genotype A when boys with AGE were considered the control group (OR = 2.2, 95% CI 0.9 to 5.1, *p* 0.05) in relation to boys with AGE (ARI control group, OR = 0.4, 95% CI 0.1 to 1.0, *p* 0.05). When genotype G boys with AGE were compared with boys with ARI, the latter had a lower susceptibility to acquiring ARI (AGE control group, OR = 0.4, 95% CI 0.1 to 1.0, *p* = 0.05) than AGE (ARI control group, OR = 2.2, 95% CI 0.9 to 5.1, *p* 0.05) (Table S1B, Supplementary Material). No statistical significance was observed regarding heterozygous AG girls (Table S1C, Supplementary Material), considering all children with AGE or ARI (Table S1D, Supplementary Material) or the different genotypes of boys and girls of the ACE *rs*4646994 polymorphism (Table S1A–D, Supplementary Material).

The combination of the ACE *rs*4646994 + ACE2 *rs*2285666 polymorphisms was statistically analyzed to investigate its association with AGE or ARI (Table S1E, Supplementary Material). Children with the II + G genotyping combination were more susceptible to acquiring AGE (OR = 2.2, 95% CI 1.1 to 4.5, *p* 0.02) than ARI (0.4, 95% CI 0.2 to 0.89, *p* = 0.02) (Table 4 and Table S1E, Supplementary Material).

### 3.4. Distribution of ACE rs4646994 and ACE2 rs2285666 Polymorphisms in the Population of Roraima State (Amazon Region)

**Figure 2:**
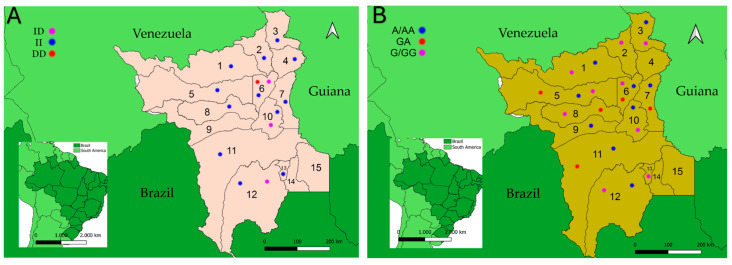
(**A**,**B**) Maps of Roraima (RR) state (the countries of Venezuela and Guiana are indicated outside of these maps), where ACE *rs*4646994 (**A**) and ACE2 *rs*22856661 (**B**) polymorphisms are indicated in small colored circles. Numbers on the Roraima map indicate each of the 15 municipalities. The names collected and corresponding numbers are 1. Amajari, 2. Pacaraima, 3. Uiramutã, 4. Normandia, 5. Alto Alegre, 6. Boa Vista, 7. Bonfim, 8. Mucajaí, 9. Iracema, 10, Cantá, 11. Caracaraí, 12. Rorainópolis, 13. São Luiz do Anauá, 14. São José da Baliza, 15. Caroebe. ^1^ Boys with genotype A were grouped with homozygous AA girls, and boys with genotype G are grouped with homozygous GG girls since the ACE2 rs22856661 polymorphism is located on the X chromosome (Xp22) in intron 3.

Children homozygous for II and heterozygous for ID were the predominant genotypes (ACE *rs*4646994) in the RR population. These genotypes were the only ones detected in 10 of the 12 RR municipalities from which the samples were collected. The municipality of Boa Vista was the only one where the three genotypes (II, ID, and DD) were detected in children and the only one where homozygous DD was also detected (Figure 2A). The genotypes of children living in the municipalities of Cantá and Rorainópolis were both II and ID (Figure 2A).

Regarding ACE2 *rs*2285666 polymorphisms, the municipalities of Boa Vista and Alto Alegre also showed children with the three genotypes (A/AA, GA, and G/GG). In other municipalities where genotyping was possible, the profiles were as diverse as A/AA and G/GG and A/AA and GA. In the municipalities of Pacaraima and Iracema, only homozygous children were detected, respectively, A/AA and G/GG (Figure 2B).

### 3.5. Very Low Frequency of SARS-CoV-2 in Children Living in the Amazon Region in the 2021 Year

Twelve children (5.9%, 12/202) had at least one of the samples (feces or saliva) collected testing positive for the SARS-CoV-2 *E* gene (Table 5); nine (4.5%, 9/202) were from children with AGE (five saliva samples and four feces samples), and three (1.5%, 3/202) from children with ARI (two saliva samples and one feces sample). Most SARS-CoV-2 positive samples were from children living in the Boa Vista municipality. The children’s ages varied, and the viral load detected in the qRT-PCR was not lower than 2.94 × 10^3^ (Table 5). No child had the SARS-CoV-2 gene detected simultaneously in the feces and the saliva samples. All positive and negative controls gave the expected results.

All 12 SARS-CoV-2 positive children were II homozygous (ACE *rs*4646994), except for one child; half were boys, and half were girls. Most girls (66.7%, 4/6) infected by SARS-CoV-2 were GA genotype (ACE2 *rs*2285666).

## 4. Discussion

This retrospective study aimed to collect samples from children living in isolated regions of the Amazon rainforest, who were treated at the only hospital in the state of RR, Brazil, prepared and structured to care for the different indigenous ethnicities that live in said areas. All the children in the municipality of Boa Vista were from ethnic groups of Macuxi, Wapixana, Taurepangue, and Yanomami, and the sample number is statistically significant, considering the studied population. HCSA cares for children who arrive mainly with AGE and ARI symptoms. Even during the pandemic period, when samples for this study were collected, many children presented AGE. Therefore, this study’s design was based on the hypothesis that AGE is associated with SARS-CoV-2 infections. In many previous studies with children infected by the SARS-CoV-2 virus, fecal shedding was accompanied by gastrointestinal symptoms such as nausea, vomiting, and diarrhea in approximately 18% (12–25%) of cases [30,31]. Indeed, despite the low frequency of SARS-CoV-2 detected by qRT-PCR in this study, the detections were more in the AGE group (8.9%) than in the ARI group (2.9%). The low frequency of SARS-CoV-2 could be related to the collection period (from May to June 2021). Recently, our group demonstrated statistically that climatic factors positively affected norovirus infections from May to June [32], when high frequencies of this virus were detected, which were considered one of the main causes of AGE. The samples in this study were collected in May and June 2021, and feces were recently examined for the presence of norovirus GI/GII by qRT-PCR. High frequencies were detected (31.7%, 64/202). Our hypothesis is that norovirus, even if using a different infection route, could somehow have contributed to the low frequency of SARS-CoV-2 detection by effectively interfering with the virus. The ACE2 SARS-CoV-2 receptor is expressed ubiquitously across various tissues, with abundant expression reported in the lungs and intestines [33]. Zenarruzabeitia et al., 2021, showed the role of the CD300 receptor in severe COVID-19 [34]; this receptor has been identified as essential for murine norovirus infection [35]. Interference has been implicated in previous pandemics and is currently not well-characterized in the setting of the COVID-19 pandemic. Recently, Deleveaux et al., 2023, concluded that influenza A could reduce SARS-CoV-2 replication [36].

The low frequency of SARS-CoV-2 detection in the children in this study prevented genetic associations with the ACE *rs*4646994 and ACE2 *rs*2285666 polymorphisms. However, it was possible to identify how genetically particular the population of this study is. For the ACE *rs*4646994 polymorphism, the high prevalence of genotype II was the opposite of studies with non-indigenous Brazilian populations, in which the DD and ID genotypes were predominant [37,38]. The allelic frequencies of the I and D genotypes within various geographic regions vary greatly [39]. Ethnic populations such as Ashkenazi Jews and Canarians present differences; differences are also shown in relation to populations of black Africans, in which the frequency of the D allele is 60%. In Oceania and Asia, there is an increase in the detection of the I allele [39]. The genotypes for the ACE *rs*4646994 polymorphism of children of this study living in the Northwest Amazon region are different from those living in other parts of the Amazon rainforest [40]. The low frequency of SARS-CoV-2 detected in the children in this study may be associated with the prevalence of genotype II since the homozygous individual DD has been statistically associated with patients with severe COVID-19 [41,42,43,44,45]. Very recently, Putira Sacuena et al., 2024, [46] identified the ACE *rs*4646994 polymorphism in 263 individuals from different indigenous ethnicities in the Amazon region aged between 10 and 95 years, with the aim of identifying the genetic factors that, in their study population, could contribute to the low frequency of SARS-CoV-2 infection detected in the Amazon during the COVID-19 pandemic. Like our study, the authors did not identify any correlation between the ACE *rs*4646994 polymorphism and the presence or absence of SARS-CoV-2 IgG antibodies. Genotype II (*rs*4646994 polymorphism) was the genotype predominantly detected, and children presenting this genotype were more susceptible to AGE than ARI, which could be considered evidence that the II genotype is not indeed associated with respiratory symptoms. The profile of the ACE2 *rs*2285666 polymorphism detected in this study population was like other studies previously presented [41,42,43]. In our study, boys with genotype A (ACE2 *rs*2285666 polymorphism) were more susceptible to symptoms of ARI and pneumonia than AGE. To our knowledge, this is the first study statistically showing the association between the ACE2 *rs*2285666 polymorphism and pneumonia unlinked to SARS-CoV-2 infection. Only one boy with the genotype A tested positive for the *E* gene of SARS-CoV-2. The detection of solely the *E* gene in this study may indicate a mild or asymptomatic profile of young children, where SARS-CoV-2 replication is not efficient [47,48]. Van Heirstraeten et al., 2022, [31] verified limited transmission of SARS-CoV-2 in Belgian daycare centers among young children aged 6–30 months during May 2020–February 2022. ACE2 *rs*2285666 polymorphism is linked to SARS-CoV-2 infection. However, different studies showed that neither the disease severity of COVID-19 nor sex influenced ACE2 levels [16,41,42,43,49,50]. Boys presenting genotype G or combined II + G were more susceptible to AGE than ARI, also confirming the genetic susceptibility of genotype A (ACE2 *rs*2285666 polymorphism) to symptoms of ARI and pneumonia. Saliva was collected for SARS-CoV-2 molecular testing because it was less invasive for such young children in this study. Furthermore, it has been shown to be a safe and feasible collection method for early and unbiased detection of SARS-CoV-2 [51,52,53]. The PCR-RFLP/*Alu*l digestion to define ACE2 *rs*2285666 polymorphism is laborious, and unfortunately, we were unable to obtain results from 18.3% of the children in this study. The SYBR Green real-time PCR to ACE *rs*4646994 polymorphism yielded 100%, proving their applicability.

## 5. Conclusions

Our study highlights the importance of genetic studies involving genetically isolated populations with unique infection profiles. It provides important data, mainly on susceptibility to ACE2 *rs*2285666 polymorphism associated with ARI and pneumonia.

## Figures and Tables

**Table 1:** Study population and sample data.

Municipality ^1^ Name	*n* ^2^ (%)	Ethnic Group ^3, 5^
Alto Alegre	10 (4.9)	Macuxi, Wapixana, and Yanomami
Amajari	6 (2.9)	Macuxi, Wapixana, and Yanomami
Boa vista ^3^	151 (75.0)	Macuxi, Wapixana, Taurepangue ^4^, and Yanomami
Bonfim	3 (1.5)	Macuxi and Wapixana
Cantá	5 (2.5	Wapixana
Caracaraí	4 (1.9)	Yanomami
Iracena	1 (0.5)	Yanomami
Mucajaí	3 (1.5)	Yanomami
Normandia	1 (0.5)	Macuxi, Wapixana, and Ingarincó
Pacaraima	5 (2.5)	Macuxi, Wapixana, and Taurepangue
Rorainópolis	6 (2.9)	Waimirim and Afroari
São Luiz do Anauá	1 (0.5)	Wai-Wai
Uiramuitã	6 (2.9)	Macuxi, Wapixana, and Ingarincó
Total	202 (100.0)	

^1^ Municipality name is the region of origin of each child tended to, and all the municipalities are in the state of Roraima (RR); no samples were collected from children living in the municipalities of São José da Baliza and Caroebe. ^2^ *n* = total number of children, from which two samples were collected (one saliva and one feces); (%) = percent of the total number of children. ^3^ Ethnic group corresponds to the group of individuals that lives in the region to which the children belong. ^4^ The Taurepang self-designate Pemon, a term that means “people”. ^5^ The ethnic group to which the children belong, as declared by their parents, does not necessarily represent their pure ethnicity. However, there is a real ancestry denoted by physical and cultural aspects.

**Table 2:** Genotypes of the ACE *rs*4646994 and ACE2 *rs*2285666 polymorphisms detected in children ≤3 years of age living in the Amazon region.

**Polymorphism** **Genotype ^1^**	**Children Symptom**					
	**AGE *n* = 101**		**ARI ^2^ *n* = 101**		**All ^3^ *n* = 202**	
	Frequency *n* (% ^4^)					
	Boys *n* = 121 63 (52.0)	Girls *n* = 81 38 (46.9)	Boys *n =* 121 58 (47.9)	Girls *n* = 81 43 (53.0%)	AGE 101 (50.0%)	ARI 101 (50.0%)
(ACE *rs*4646994)	Frequency *n* (% ^5^)					
II	55 (87.4)	35 (92.1)	49 (84.5)	37 (86.0)	90 (89.1)	86 (85.1)
ID	4 (6.3)	1 (2.6)	4 (6.9)	6 (14.0)	5 (4.9)	10 (9.9)
DD	4 (6.3)	2 (5.3)	5 (8.6)	None	6 (5.9)	5 (5.0)
Total	63 (100.0)	38 (100.0)	58 (100.0)	43 (100.00)	101 (100.00)	101 (100.0)
(ACE2 *rs*2285666)	Frequency *n* (%)					
A	18 (28.6)	None	22 (37.9)	None	18 (17.8)	22 (21.8)
G	37 (58.7)	None	20 (34.5)	None	37 (36.6)	20 (19.8)
AA	None	6 (15.8)	None	8 (18.6)	6 (5.9)	8 (7.9)
AG	None	11 (28.9)	None	7 (16.3)	11 (10.9)	7 (6.9)
GG	None	15 (39.5)	None	21 (48.8)	15 (14.9)	21 (20.8)
Indeterminate	8 (12.7)	6 (15.8)	16 (27.6)	7 (16.3)	14 (13.9)	23 (22.8)
Total	63 (100.0)	38 (100.0)	58 (100.0)	43 (100.00)	101 (100.00)	101 (100.00)

^1^ OR was calculated with AGE and ARI according to ACE *rs*4646994 and ACE2 *rs*2285666 genotypes detected in the children (Table S1A–D, Supplementary Material). ^2^ All boys with ARI had pneumonia. ^3^ All children (boys and girls with AGE or ARI). ^4^ Frequency calculated considering the total number of boys or/and girls with AGE or ARI. ^5^ Frequency calculated considering the number of boys with AGE or/and ARI. Abbreviations: ACE = angiotensin-converting enzyme; AGE = acute gastroenteritis; ARI = acute respiratory infection; *n* = total number of children.

**Table 3:** Genotypes of ACE2 *rs*2285666 polymorphism detected in young children with and without pneumonia in the Amazon region.

Genotype	Children ^1^ Symptom		Odds Ratio ^2^
	With Pneumonia *n* = 89	Without Pneumonia *n* = 113	
	Frequency *n* ^3^		
II	74	102	0.5 (95% CI 0.2 to 1.2, *p* 0.1)
ID	10	5	2.7 (95% CI 0.8 to 8.3, *p* 0.07)
DD	5	6	1.0 (95% CI 0.3 to 3.5, *p* 0.9)
Total	89	113	

^1^ Total of children = 202. ^2^ Odds Ratio was calculated using https://www.medcalc.org/calc/odds_ratio.php (accessed on 21 October 2024) ^3^ Frequency was calculated considering the total number of boys + girls with or without pneumonia. Abbreviations: ACE = angiotensin-converting enzyme; CI = confidence interval; *p* = probability value; *n* = total number of children.

**Table 4:** Frequency of combinations of genotypes ACE *rs*4646994 and ACE2 *rs*2285666 polymorphisms detected in young children living in the Amazon region.

Genotyping Combination ^1^ ACE *rs*4646994 + ACE2 *rs*2285666	Children ^2^ Symptom	
	AGE = *n* ^3^ (%)	ARI = *n* (%)
II + A	18 (20.7)	19 (24.3)
II + G	32 (36.8)	16 (20.5)
II + AA	6 (6.9)	6 (7.7)
II + AG	8 (9.2)	7 (9.0)
II + GG	15 (17.2)	18 (23.0)
ID + A	None	1 (1.3)
ID + G	2 (2.3)	2 (2.6)
ID + AA	None	2 (2.6)
ID + AG	1 (1.1)	None
ID + GG	None	3 (3.8)
DD + A DD + G	None 3 (3.5)	2 (2.6) 2 (2.6)
DD + AA	None	None
DD + AG DD + GG	2 (2.3) None	None None
Total	87 (100%)	78 (100%)

^1^ OR was calculated with AGE and ARI according to ACE *rs*4646994 and ACE2 *rs*2285666 genotypes detected in the children (Table S1E, Supplementary Material). ^2^ Total number of children = 202. ^3^ *n* = total number of children with a combination of ACE *rs*4646994 + ACE2 *rs*2285666; (%) = per cent of the total number of children. Abbreviations: ACE = angiotensin-converting enzyme; AGE = acute gastroenteritis; ARI = acute respiratory infection; OR = odds ratio.

**Table 5:** Characteristics of SARS-CoV-2 positive samples collected from children living in the Amazon region.

Sample ID ^1^	Sample Type	Clinical Symptoms ^2^	Age (Gender)	Viral Load ^3^ (Copies/mL)	Polimorphism Genotype		Municipality ^1^ Name
					ACE *rs*4646994	ACE *rs*2285666	
32198	feces	ARI	2 mos (Boy)	3.16 × 10^4^	II	A	Boa Vista
32200	feces	AGE	4 mos (Boy)	8.39 × 10^4^	II	G	Boa Vista
32229	saliva	AGE	16 mos (Girl)	1.43 × 10^5^	II	GA	Boa Vista
32344	feces	AGE	20 mos (Girl)	8.22 × 10^5^	ID	GA	Boa Vista
32414	feces	ARI	2 mos (Girl)	8.51 × 10^4^	II	GA	Boa Vista
32416	feces	AGE	4 mos (Boy)	2.94 × 10^3^	II	A	Boa Vista
32477	saliva	AGE	3 yrs, 9 mos (Girl)	3.71 × 10^4^	II	GA	Alto Alegre
32489	saliva	AGE	2 mos (Boy)	3.53 × 10^4^	II	A	Caracaraí
32512	feces	AGE	2 yrs, 4 mos (Boy)	1.67 × 10^5^	II	G	Boa Vista
32517	saliva	AGE	2 yrs, 5 mos (Girl)	2.62 × 10^4^	II	GG	Boa Vista
32529	saliva	AGE	7 mos (Boy)	2.73 × 10^4^	II	Indeterminate	Boa Vista
32531	saliva	ARI	10 mos (Girl)	4.38 × 10^4^	II	AA	Boa Vista

^1^ ID = laboratory identification number. ^2^ Clinical symptoms are AGE = acute gastroenteritis or ARI = acute respiratory infection. ^3^ Viral load/SARS-CoV-2, gene *E*. Abbreviations: ACE = angiotensin-converting enzyme; mos = months; yrs = years.

## Data Availability

Data are contained within the article and Supplementary Materials.

## Supplementary Materials

The following supporting information can be downloaded at https://www.mdpi.com/article/10.3390/tropicalmed9110270/s1, Table S1. (A–E). Information from odds ratio (OR) calculations. OR was done using https://www.medcalc.org/calc/odds_ratio.php. The program reports the OR with its 95% confidence interval and associated *p* (Probability) value. If *p* is less than 0.05 it can be concluded that the odds ratio is significantly different from 1 and that the odds in one group are significantly higher than in the other. AGE = Acute Gastroenteritis, ARI = Acute Respiratory Infection and CI = Confidence Interval. Light pink boxes are significant values.
