# Supplementary material for: Polymorphisms in the ACE I/D (rs4646994) and ACE2 G8790A (rs2285666) in Young Children Living in the Amazon Region and SARS-CoV-2 Infection
Source: Trop Med Infect Dis. 2024 Nov 7;9(11):270. doi: 10.3390/tropicalmed9110270 (PMC11598624; doi:10.3390/tropicalmed9110270)
Supplement: Supplementary file 1 [file tropicalmed-09-00270-s001.zip › tropicalmed-3194239-supplementary.pdf]

Supplementary Material

Table S1A-E. Information from odds ratio (OR) calculations. OR was done using https://www.medcalc.org/calc/odds_ratio.php. The program reports the OR with its 95% confidence interval and associated *P* (Probability) value. If *P* is less than 0.05 it can be concluded that the odds ratio is significantly different from 1 and that the odds in one group are significantly higher than in the other. AGE= Acute Gastroenteritis, ARI = Acute Respiratory Infection and CI = Confidence Interval. Light pink boxes are significant values.

A. Odds Ratio calculation – outcome with ACE rs4646994 or ACE2 rs2285666 polymorphisms (girls or boys as control groups)

Polymorphism	Clinical symptoms			
	AGE		ARI	
	Boys (N)	Girls (N)	Boys (N)	Girls (N)
**ACE rs4646994**				
N1= genotyped	63	38	58	43
II	**55**	**35**	**49**	**37**
Total 1 (N1 - II)	**8**	**3**	**9**	**6**
OR= (axd)/(bxc)	55/8/35/3	35/3/55/8	49/9/37/6	37/6/49/9
OR (CI, *P*), boys with bad outcomes AGE	0.5893 (95 % CI: 0.1463 to 2.3729) P = 0.4568			
OR (CI, *P*), boys with bad outcomes ARI			0.8829 (95 % CI: 0.2887 to 2.6998) P = 0.8271	
OR (CI, *P*), girls with bad outcomes AGE	1.6970 (95 % CI: 0.4214 to 6.8333) P = 0.4568			
OR (CI, *P*), girls with bad outcomes ARI			1.1327 (95 % CI: 0.3704 to 3.4636) P = 0.8271	
ID	**4**	**1**	**4**	**6**
Total 1 (N1 - II)	**59**	**37**	**54**	**37**
OR= (axd)/(bxc)	4/59/1/37	1/37/4/59	4/54/6/37	6/37/4/54
OR (CI, *P*), boys with bad outcomes AGE	2.5085 (95 % CI: 0.2699 to 23.3182) P = 0.4188			
OR (CI, *P*), boys with bad outcomes ARI			0.4568 (95 % CI: 0.1205 to 1.7315) P = 0.2491	
OR (CI, *P*), girls with bad outcomes AGE	0.3986 (95 % CI: 0.0429 to 3.7057) P = 0.4188			
OR (CI, *P*), girls with bad outcomes ARI			2.1892 (95 % CI: 0.5775 to 8.2984) P = 0.2491	
DD	**4**	**2**	**5**	**0**
Total 1 (N1 - DD)	**59**	**36**	**53**	**43**
OR=(axd)/(bxc)	4/59/2/36	2/36/4/59	5/53/0/43	0/43/5/53
OR (CI, *P*), boys with bad outcomes AGE	1.2203 (95 % CI: 0.2126 to 7.0036) P = 0.8232			
OR (CI, *P*), boys with bad outcomes ARI			8.9439 (95 % CI: 0.4811 to 166.2693) P = 0.1417	
OR (CI, *P*), girls with bad outcomes AGE	0.8194 (95 % CI: 0.1428 to 4.7028) P = 0.8232			
OR (CI, *P*), girls with bad outcomes ARI			0.1118 (95 % CI: 0.0060 to 2.0785) P = 0.1417	
**ACE2 rs2285666**				
N1= submitted to genotyping	63	38	58	43
N2= not genotyped	8	6	16	7
Total 1 = Genotyped (N1 - N2)	55	32	42	36
A/AA = N	**18**	**6**	**22**	**8**
Total 2 (Total 1 – A/AA)	**37**	**26**	**20**	**28**
OR=(axd)/(bxc)	18/37/6/26	6/26/18/37	22/20/8/28	8/28/22/20
OR (CI, *P*), boys with bad outcomes AGE	2.1081 (95 % CI: 0.7367 to 6.0322) P = 0.1644			
OR (CI, *P*), boys with bad outcomes ARI			3.8500 (95 % CI: 1.4277 to 10.3822) P = 0.0077	
OR (CI, *P*), girls with bad outcomes AGE	0.4744 (95 % CI: 0.1658 to 1.3573) P = 0.1644			
OR (CI, *P*), girls with bad outcomes ARI			0.2597 (95 % CI: 0.0963 to 0.7004) P = 0.0077	
G/GG = N	**37**	**15**	**20**	**21**
Total 2 (Total 1 – G/GG)	**18**	**17**	**22**	**15**
OR=(axd)/(bxc)	37/18/15/17	15/17/37/18	20/22/21/15	21/15/20/22
OR (CI, *P*), boys with bad outcomes AGE	2.3296 (95 % CI: 0.9528 to 5.6960) P = 0.0637			
OR (CI, *P*), boys with bad outcomes ARI			0.6494 (95 % CI: 0.2646 to 1.5934) P = 0.3458	
OR (CI, *P*), girls with bad outcomes AGE	0.4293 (95 % CI: 0.1756 to 1.0495) P = 0.0637			
OR (CI, *P*), girls with bad outcomes ARI			1.5400 (95 % CI: 0.6276 to 3.7788) P = 0.3458	

B. Odds Ratio calculation – outcome with ACE rs4646994 or ACE2 rs2285666 polymorphisms (AGE or ARI as control groups)

**Polymorphism**	**Clinical symptoms**			
	**AGE**	**ARI**	**AGE**	**ARI**
	Boys (N)	Boys (N)	Girls (N)	Girls (N)
**ACE rs4646994**				
N1= genotyped	63	58	38	43
II	**55**	**49**	**35**	**37**
Total 1 (N1 - II)	**8**	**9**	**3**	**6**
OR=(axd)/(bxc)	55/8/49/9	49/9/55/8	35/3/37/6	37/6/35/3
OR (CI, *P*), boys with bad outcomes AGE	1.2628 (95 % CI: 0.4520 to 3.5274) P = 0.6562			
OR (CI, *P*), boys with bad outcomes ARI	0.7919 (95 % CI: 0.2835 to 2.2122) P = 0.6562			
OR (CI, *P*), girls with bad outcomes AGE			1.8919 (95 % CI: 0.4389 to 8.1542) P = 0.3923	
OR (CI, *P*), girls with bad outcomes ARI			0.5286 (95 % CI: 0.1226 to 2.2782) P = 0.3923	
ID	**4**	**4**	**1**	**6**
Total 1 (N1 - II)	**59**	**54**	**37**	**37**
OR=(axd)/(bxc)	4/59/4/54	4/54/4/59	1/37/6/37	6/37/1/37
OR (CI, *P*), boys with bad outcomes AGE	0.9153 (95 % CI: 0.2181 to 3.8408) P = 0.9037			
OR (CI, *P*), boys with bad outcomes ARI	1.0926 (95 % CI: 0.2604 to 4.5850) P = 0.9037			
OR (CI, *P*), girls with bad outcomes AGE			0.1667 (95 % CI: 0.0191 to 1.4532) P = 0.1049	
OR (CI, *P*), girls with bad outcomes ARI			6.0000 (95 % CI: 0.6881 to 52.3153) P = 0.1049	
DD	**4**	**5**	**2**	**0**
Total 1 (N1 - DD)	**59**	**53**	**36**	**43**
OR=(axd)/(bxc)	4/59/5/53	5/53/4/59	2/36/0/43	0/43/2/36
OR (CI, *P*), boys with bad outcomes AGE	0.7186 (95 % CI: 0.1833 to 2.8172) P = 0.6355			
OR (CI, *P*), boys with bad outcomes ARI	1.3915 (95 % CI: 0.3550 to 5.4549) P = 0.6355			
OR (CI, *P*), girls with bad outcomes AGE			5.9589 (95 % CI: 0.2771 to 128.1259) P = 0.2542	
OR (CI, *P*), girls with bad outcomes ARI			0.1678 (95 % CI: 0.0078 to 3.6083) P = 0.2542	
**ACE2 rs2285666**				
N1= submitted to genotyping	63	58	38	43
N2= not genotyped	8	16	6	7
Total 1 = Genotyped (N1 - N2)	55	42	32	36
A/AA = N	**18**	**22**	**6**	**8**
Total 2 (Total 1 – A/AA)	**37**	**20**	**26**	**28**
OR=(axd)/(bxc)	18/37/22/20	22/20/18/37	6/26/8/28	8/28/6/26
OR (CI, *P*), boys with bad outcomes AGE	0.4423 (95 % CI: 0.1934 to 1.0112) P = 0.0532			
OR (CI, *P*), boys with bad outcomes ARI	2.2611 (95 % CI: 0.9889 to 5.1700) P = 0.0532			
OR (CI, *P*), girls with bad outcomes AGE			0.8077 (95 % CI: 0.2468 to 2.6430) P = 0.7240	
OR (CI, *P*), girls with bad outcomes ARI			1.2381 (95 % CI: 0.3784 to 4.0515) P = 0.7240	
G/GG = N	**37**	**20**	**15**	**21**
Total 2 (Total 1 – G/GG)	**18**	**22**	**17**	**15**
OR=(axd)/(bxc)	37/18/20/22	20/22/37/18	15/17/21/15	21/15/15/17
OR (CI, *P*), boys with bad outcomes AGE	2.2611 (95 % CI: 0.9889 to 5.1700) P = 0.0532			
OR (CI, *P*), boys with bad outcomes ARI	0.4423 (95 % CI: 0.1934 to 1.0112) P = 0.0532			
OR (CI, *P*), girls with bad outcomes AGE			0.6303 (95 % CI: 0.2414 to 1.6456) P = 0.3458	
OR (CI, *P*), girls with bad outcomes ARI			1.5867 (95 % CI: 0.6077 to 4.1429) P = 0.3458	

C. Odds Ratio calculation – outcome from heterozygous girls' with ACE2 rs2285666 polymorphism (AGE or ARI as control groups)

**Polimorphism ACE2 rs2285666**	**Clinical symptoms**	
	**AGE**	**ARI**
	Girls (N)	Girls (N)
N1= submitted to genotyping	38	43
N2= not genotyped	6	7
Total 1 = Genotyped (N1 - N2)	32	36
AG = N	**11**	**7**
Total 2 (Total 1 – AG)	**21**	**29**
OR=(axd)/(bxc)	11/21/7/29	7/29/11/21
OR (CI, *P*), girls with bad outcomes AGE	2.1701 (95 % CI: 0.7212 to 6.5294) P = 0.1680	
OR (CI, *P*), girls with bad outcomes ARI	0.4608 (95 % CI: 0.1532 to 1.3865) P = 0.1680	

D. Odds Ratio calculation – outcome with ACE rs4646994 or ACE2 rs2285666 polymorphisms, with the total number of girls and boys (AGE or ARI as control groups)

**Polimorphism ACE2 rs2285666**	**Clinical symptoms**	
	**AGE**	**ARI**
	Boys + Girls (N)	Boys + Girls (N)
**ACE rs4646994**		
N1= genotyped	101	101
II	**90**	**86**
Total 1 (N1 - II)	**11**	**15**
OR=(axd)/(bxc)	90/11/86/15	86/15/90/11
OR (CI, *P*), Boys + girls with bad outcomes AGE	1.4271 (95 % CI: 0.6208 to 3.2802) P = 0.4023	
OR (CI, *P*), Boys + girls with bad outcomes ARI		0.7007 (95 % CI: 0.3049 to 1.6107) P = 0.4023
ID	**5**	**10**
Total 1 (N1 - ID)	**96**	**91**
OR=(axd)/(bxc)	5/96/10/91	10/91/5/96
OR (CI, *P*), Boys + girls with bad outcomes AGE	0.4740 (95 % CI: 0.1560 to 1.4399) P = 0.1878	
OR (CI, *P*), Boys + girls with bad outcomes ARI		2.1099 (95 % CI: 0.6945 to 6.4097) P = 0.1878
DD	**6**	**5**
Total 1 (N1 - DD)	**95**	**96**
OR=(axd)/(bxc)	6/95/5/96	5/96/6/95
OR (CI, *P*), Boys + girls with bad outcomes AGE	1.2126 (95 % CI: 0.3579 to 4.1085) P = 0.7568	
OR (CI, *P*), Boys + girls with bad outcomes ARI		0.8247 (95 % CI: 0.2434 to 2.7940) P = 0.7568
**ACE2 rs2285666**		
N1= submitted to genotyping	101	101
N2= not genotyped	14	23
Genotyped (N1 - N2)	87	78
A/AA = N	**24**	**30**
Total 2 (Total 1 – AA)	**63**	**48**
OR=(axd)/(bxc)	24/63/30/48	30/48/24/63
OR (CI, *P*), Boys + girls with bad outcomes AGE	0.6095 (95 % CI: 0.3166 to 1.1735) P = 0.1385	
OR (CI, *P*), Boys + girls with bad outcomes ARI		1.6406 (95 % CI: 0.8521 to 3.1587) P = 0.1385
G/GG = N	**52**	**41**
Total 2 (Total 1 – GG)	**35**	**37**
OR=(axd)/(bxc)	52/35/41/37	41/37/52/35
OR (CI, *P*), Boys + girls with bad outcomes AGE	1.3408 (95 % CI: 0.7231 to 2.4859) P = 0.3519	
OR (CI, *P*), Boys + girls with bad outcomes ARI		0.7458 (95 % CI: 0.4023 to 1.3828) P = 0.3519

E. Odds Ratio calculation – outcome with ACE rs4646994+ACE2 rs2285666 polymorphisms combinations[1], with the total number of girls and boys (AGE or ARI as control groups)

**Polimorphism ACE2 rs2285666**	**Clinical symptoms**	
	**AGE**	**ARI**
	Boys and/or Girls (N)	Boys and/or Girls (N)
**ACE rs4646994 + ACE2 rs2285666**		
N1= genotyped	87	78
II+A	**18**	**19**
Total 1 (N1 – II+A)	**69**	**59**
OR=(axd)/(bxc)	18/69/19/59	19/59/18/69
OR (CI, *P*), Boys with bad outcomes AGE	0.8101 (95 % CI: 0.3894 to 1.6850) P = 0.5730	
OR (CI, *P*), Boys with bad outcomes ARI		1.2345 (95 % CI: 0.5935 to 2.5677) P = 0.5730
II+G	**32**	**16**
Total 1 (N1 – II+G)	**55**	**62**
OR=(axd)/(bxc)	32/55/16/62	16/62/32/55
OR (CI, *P*), Boys with bad outcomes AGE	2.2545 (95 % CI: 1.1180 to 4.5464) P = 0.0231	
OR (CI, *P*), Boys with bad outcomes ARI		0.4435 (95 % CI: 0.2200 to 0.8944) P = 0.0231
II+AA	**6**	**6**
Total 1 (N1 – II+AA)	**81**	**77**
OR=(axd)/(bxc)	6/81/6/77	6/77/6/81
OR (CI, *P*), Girls with bad outcomes AGE	0.9506 (95 % CI: 0.2939 to 3.0746) P = 0.9326	
OR (CI, *P*), Girls with bad outcomes ARI		1.0519 (95 % CI: 0.3252 to 3.4023) P = 0.9326

II+AG	**8**	**7**
Total 1 (N1 – II+AG)	**79**	**71**
OR=(axd)/(bxc)	8/79/7/71	7/71/8/79
OR (CI, *P*), Girls with bad outcomes AGE	1.0271 (95 % CI: 0.3545 to 2.9760) P = 0.9607	
OR (CI, *P*), Girls with bad outcomes ARI		0.9736 (95 % CI: 0.3360 to 2.8209) P = 0.9607
II+GG	**15**	**18**
Total 1 (N1 – II+GG)	**72**	**60**
OR=(axd)/(bxc)	15/72/18/60	18/60/15/72
OR (CI, *P*), Girls with bad outcomes AGE	0.6944 (95 % CI: 0.3228 to 1.4940) P = 0.3509	
OR (CI, *P*), Girls with bad outcomes ARI		1.4400 (95 % CI: 0.6693 to 3.0980) P = 0.3509
ID+G	**2**	**2**
Total 1 (N1 – ID+G)	**85**	**76**
OR=(axd)/(bxc)	2/85/2/76	2/76/2/85
OR (CI, *P*), Boys with bad outcomes AGE	0.8941 (95 % CI: 0.1229 to 6.5036) P = 0.9120	
OR (CI, *P*), Boys with bad outcomes ARI		1.1184 (95 % CI: 0.1538 to 8.1351) P = 0.9120
ID+GG	**1**	**3**
Total 1 (N1 – ID+GG)	**86**	**75**
OR=(axd)/(bxc)	1/86/3/75	3/75/1/86
OR (CI, *P*), Girls with bad outcomes AGE	0.2907 (95 % CI: 0.0296 to 2.8543) P = 0.2891	
OR (CI, *P*), Girls with bad outcomes ARI		3.4400 (95 % CI: 0.3503 to 33.7767) P = 0.2891
DD+G	**3**	**2**
Total 1 (N1 – DD+G)	**84**	**76**
OR=(axd)/(bxc)	3/84/2/76	2/76/3/84
OR (CI, *P*), Boys with bad outcomes AGE	1.3571 (95 % CI: 0.2208 to 8.3421) P = 0.7417	
OR (CI, *P*), Boys with bad outcomes ARI		0.7368 (95 % CI: 0.1199 to 4.5292) P = 0.7417

[1] Polymorphism combinations ID+A; ID+AA, ID+AG, DD+A, DD+AA, DD+AG and DD+GG were excluded from OR calculations because no control group to be selected existed.
